# The Role of Nrf2 in Pulmonary Fibrosis: Molecular Mechanisms and Treatment Approaches

**DOI:** 10.3390/antiox11091685

**Published:** 2022-08-29

**Authors:** Yu Wang, Juan Wei, Huimin Deng, Li Zheng, Hao Yang, Xin Lv

**Affiliations:** 1 Department of Anesthesiology, The First Affiliated Hospital of Anhui Medical University, Hefei 230022, China; 2 Department of Anesthesiology, Shanghai Pulmonary Hospital, School of Medicine, Tongji University, Shanghai 200433, China

**Keywords:** nuclear factor erythroid 2-related factor 2, pulmonary fibrosis, inflammation, oxidative stress, signaling pathways

## Abstract

Pulmonary fibrosis is a chronic, progressive, incurable interstitial lung disease with high mortality after diagnosis and remains a global public health problem. Despite advances and breakthroughs in understanding the pathogenesis of pulmonary fibrosis, there are still no effective methods for the prevention and treatment of pulmonary fibrosis. The existing treatment options are imperfect, expensive, and have considerable limitations in effectiveness and safety. Hence, there is an urgent need to find novel therapeutic targets. The nuclear factor erythroid 2-related factor 2 (Nrf2) is a central regulator of cellular antioxidative responses, inflammation, and restoration of redox balance. Accumulating reports reveal that Nrf2 activators exhibit potent antifibrosis effects and significantly attenuate pulmonary fibrosis in vivo and in vitro. This review summarizes the current Nrf2-related knowledge about the regulatory mechanism and potential therapies in the process of pulmonary fibrosis. Nrf2 orchestrates the activation of multiple protective genes that target inflammation, oxidative stress, fibroblast–myofibroblast differentiation (FMD), and epithelial–mesenchymal transition (EMT), and the mechanisms involve Nrf2 and its downstream antioxidant, Nrf2/HO−1/NQO1, Nrf2/NOX4, and Nrf2/GSH signaling pathway. We hope to indicate potential for Nrf2 system as a therapeutic target for pulmonary fibrosis.

## 1. Introduction

Pulmonary fibrosis (PF) describes a heterogeneous group of chronic, progressive, and incurable interstitial lung disorders characterized by induced scar formation and irreversible destruction of the lung parenchyma [1,2,3,4]. Based on etiological factors, fibrotic lung diseases are mainly classified into idiopathic pulmonary fibrosis (IPF), allergic asthma, cystic lung disease, scleroderma, granulomatous lung disease, sarcoidosis, and chronic obstructive pulmonary disease (COPD) [5]. Among these, IPF is the most notable and common type of idiopathic interstitial pneumonia, lacking an identifiable etiology and with high mortality [1,6]. It is characterized by aberrantly activated lung epithelial cells, inflammatory infiltrate, activation of lung fibroblasts, and excessive accumulation of extracellular matrix (ECM) in lung tissues that ultimately lead to respiratory failure, and eventually death if left untreated [7,8]. An array of triggers, including environmental pollutants, herbicides, drug side effects, particles, genetic abnormalities, autoimmune disorders, chronic infection, and cigarette smoking may cause IPF [9,10,11,12]. Nearly 200,000 people in the United States and over 5 million people worldwide are affected by IPF, and approximately 82–83% of deaths, incident cases, and prevalent cases occur in patients over 70 years old, imposing a great economic burden on the country as well as individuals [13,14,15]. A recent study from Germany showed that most patients with IPF did not receive medication [13], but previous research has demonstrated that patients who develop IPF without being treated have a median survival of only 3–5 years after diagnosis [3]. Consequently, there remains a major medical need for effective, safe, and well-tolerated treatments for IPF.

**Figure 1:**
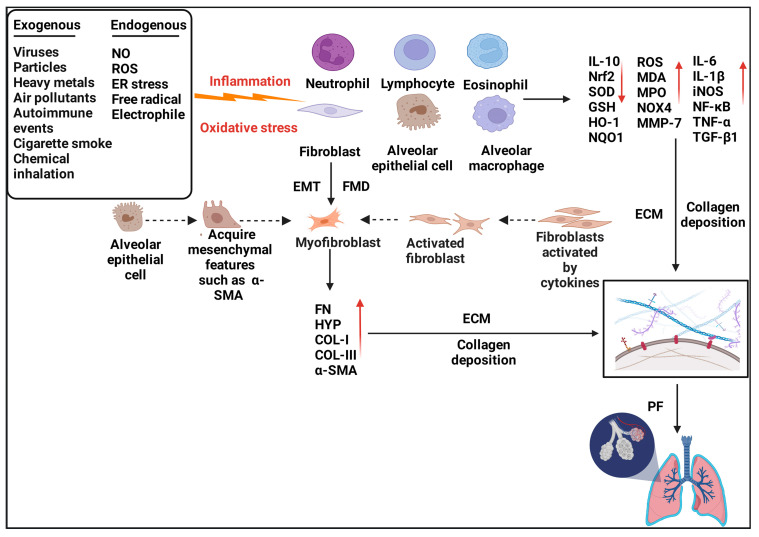
**Pathogenesis of pulmonary fibrosis.** Various exogenous and endogenous factors, such as autoimmune diseases, viral infections, cigarette smoke, inhalation of toxic substances, and free radicals, can lead to damage to the alveolar epithelial cells (AECs), aberrant activation of immune cells (neutrophils, alveolar macrophages, lymphocytes, and eosinophils), and fibroblasts. These lung cells secrete multiple pro-inflammatory and pro-fibrotic factors which can accelerate the EMT process, induce the transition of AECs to lung fibroblasts, trigger the activation of quiescent fibroblasts, and promote the differentiation of fibroblasts to myofibroblasts. Myofibroblasts can further release a large amount of collagen and ECM, resulting in hyperproliferation of fibroblasts and accelerates the progression of pulmonary fibrosis.

Unfortunately, there is currently no effective treatment for curing or reversing the progression of PF. Immunosuppressants (e.g., cyclophosphamide) and corticosteroids (e.g., dexamethasone) have been used to treat acute exacerbation of IPF, aiming at reducing symptoms and the underlying inflammation, but limited efficacy and potential side effects have restricted their application [16,17]. The FDA approved the anti-fibrotic drugs nintedanib and pirfenidone for the treatment of PF in 2014 because they were shown to delay the progression of PF [18,19]. However, while they show a clinical benefit, they cannot improve survival [20]. Currently, lung transplantation is the only life-sustaining intervention for end-stage IPF [21], but chronic lung transplant dysfunction, infection, and extrapulmonary complications lead to a poor postoperative long-term survival rate [22]. The high cost of operation and the scarcity of lung donors are two specific challenges that require consideration. Even though the pathogenic mechanisms of IPF having been studied extensively (Figure 1), few therapeutics have been successfully used in the clinic, and potential treatment methods to improve patients’ quality of life are lacking [23].

Therefore, clarifying the underlying molecular mechanism in PF and discovering efficient prevention and treatment approaches that could increase life expectancy are urgent future research directions.

## 2. Nrf2

Extensive studies have demonstrated that the nuclear factor erythroid 2-related factor 2 (Nrf2) is a critical transcription factor that coordinates the expression of more than 500 cytoprotective and metabolic genes [24], particularly classic antioxidant and detoxification enzymes, to restore internal cellular homeostasis, redox balance, and the response to diverse stresses [25,26,27]. It is now widely recognized that Nrf2 plays a protective role in many diseases in multiple organ systems, such as osteoporosis [28], Alzheimer’s disease [29], lung fibrosis [30], kidney [31], and cardiovascular system disease [32], in which oxidative stress and inflammation are thought to participate in the underlying pathological mechanisms.

### 2.1. Structure of Nrf2 and Keap1

**Figure 2:**
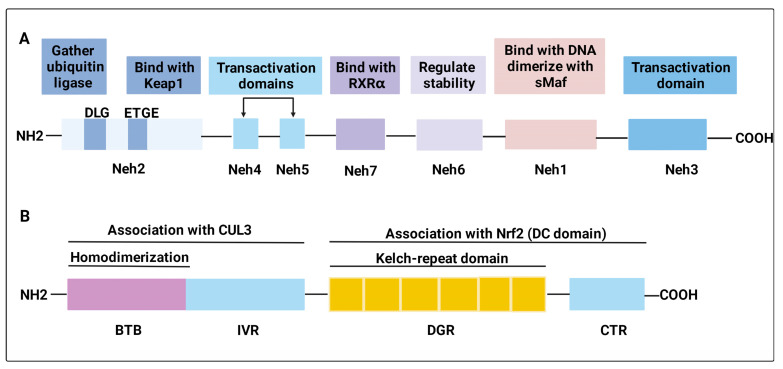
**Structures of Nrf2 and Keap1.** (**A**) The Nrf2 protein comprises seven Neh domains, known as Neh1–Neh7. The Neh1 domain is responsible for DNA binding and dimerization with the sMaf proteins; the Neh2 domain mediates the interaction with Keap1 through the ETGE motifs and gathers a ubiquitin ligase to the fusion protein through the DLG element; the Neh3, Neh4, and Neh5 domains are transactivation domains; the Neh6 domain regulates Nrf2 stability; and the Neh7 domain binds with RXRα to weaken the cytoprotective effect of Nrf2. (**B**) Keap1 possesses three functional domains. The BTB domain mediates Keap1 homodimerization and associates with Cul3; the DC directly associates with Nrf2 Neh2 domain; IVR domain contains critical cysteine residues and connects the BTB domain with DC domain.

Nrf2, first isolated and characterized by Moi et al. in 1994, is encoded by the Nuclear Factor, Erythroid 2 Like 2 (NFE2) gene and belongs to the Cap“n” Collar (CNC) subfamily of basic leucine zipper (bZIP) transcription factors [33]. Nrf2 contains seven highly conserved Nrf2 ECH homology (Neh) domains, each with a distinct function, known as Neh1–Neh7 (Figure 2A) [34]. Among these, the Neh2 domain, which is located in the N-terminus of Nrf2, plays a major regulatory role. Neh2 harbors two separate sequences, the DLG element and the ETGE tetrapeptide, mediating the process of gathering a ubiquitin ligase to the fusion protein and the redox-sensitive recruitment of Nrf2 to Kelch-like ECH-associated protein 1 (Keap1), respectively [35,36,37]. Notably, the ETGE motif is the Keap1-binding site. Neh1 contains the DNA binding motif and the Cap“n” collar basic leucine zipper domain that dimerizes with small Maf proteins on the promoters of target genes. It has been reported to regulate the stability of Nrf2 by forming a nuclear complex with UbcM2, a ubiquitin-conjugating enzyme [38]. Neh3, Neh4, and Neh5 are transactivation domains that interact with coactivators [39,40]. Neh6 is a Keap1-independent degron of Nrf2. It harbors a group of serine residues phosphorylated by glycogen synthase kinase 3 (GSK-3), resulting in the facilitation of Nrf2 degradation and regulating the stability of Nrf2 [41,42]. Previous evidence has shown that Neh7 binds with retinoic X receptor alpha (RXRα) to weaken the cytoprotective effect of Nrf2 [43].

### 2.2. Nrf2 Activation

**Figure 3:**
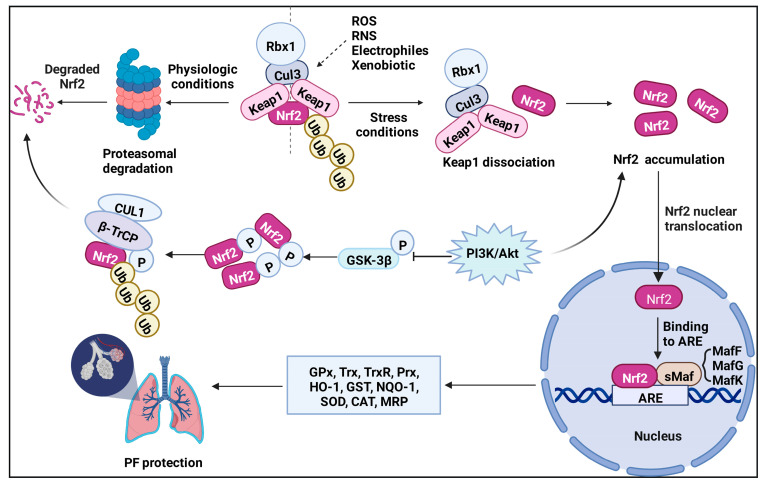
**Mechanism of Nrf2 activation.** Under normal physiological conditions, Nrf2 is sequestered in the cytoplasm by its physical interaction with Keap1 and is degraded by the ubiquitin-proteasome in the Keap1-dependent manner or the Keap1-independent manner (GSK-3β pathway). In the stress conditions, Keap1 is inactivated while Nrf2 is stabilized. The stabilized Nrf2 performs nuclear translocation and heterodimerizes with sMaf to activate target genes for cell protection through ARE. Nrf2 target genes include GPx, Trx, TrxR, Prx, HO−1, GST, NQO1, SOD, and CAT which defend lungs from further damages. Furthermore, Nrf2 also can be directly phosphorylated by GSK-3β, enabling its recognition by β-TRCP, which later marks Nrf2 for ubiquitination and degradation through the proteasome. However, PI3K/AKT signaling pathway can suppress GSK-3β by AKT-mediated phosphorylation and causing Nrf2 accumulation in the cytoplasm. The accumulation of Nrf2 further activates transcription of a number of cytoprotective genes and protection against pulmonary fibrosis.

Under a normal physiologic state, Nrf2 is anchored in the cytoplasm by binding to the E3 ubiquitin ligase Keap1 or phosphorylated by glycogen synthase kinase 3β (GSK-3β), targeting Nrf2 for its ubiquitination and proteasomal degradation [44,45]. Keap1 is a component of a ubiquitin E3 ligase [46], acting as a cysteine thiol-rich sensor of redox insults [47]. In 1999, Keap1 was identified by analyzing differential Nrf2 activity manifested in a transfected cell line [36]. Keap1 possesses three functional domains, a broad complex/tram track/bric-a-brac (BTB), an intervening region (IVR), and a double glycine repeat (DGR) and COOH-terminal region (CTR) domain (DC domain) (Figure 2B). Keap1 associates with CUL3 through its BTB domain, which is required for Keap1 dimerization [48], and part of its intervening region (IVR) domain [49,50]. The Nrf2 Neh2 domain directly interacts with the Keap1 DC domain [51]. Keap1, Cul3, and Rbx1 assemble into an efficient E3 ubiquitin ligase, which negatively regulates Nrf2 protein levels [52]. Nrf2 is ubiquitously expressed in all cell types, but its expression is usually maintained at a low level under basal conditions because the Nrf2 protein is synthesized but constantly degraded. However, upon exposure to oxidative and xenobiotic stresses, cysteine residues on Keap1, which have high redox sensitivity, can easily be covalently modified or oxidized, resulting in a conformational change and the dissociation of Nrf2 [49]. Another Keap1-independent mechanism is to escape GSK-3β-mediated degradation [44,53]. Nrf2 is phosphorylated by GSK-3β, making it identifiable by β-transducin repeat-containing protein (β-TRCP), which later marks Nrf2 for ubiquitination and degradation through the proteasome [54,55]. Meanwhile, several investigators have found that Nrf2 signaling can be activated through the PI3K-AKT signaling pathway [56,57], because GSK-3β can be inhibited by AKT-mediated phosphorylation. Subsequently, Nrf2 degradation is stagnated, leading to a rapid accumulation of Nrf2 in the cytoplasm and translocation to the nucleus. In the nucleus, Nrf2 heterodimerizes with small v-maf avian musculoaponeurotic fibrosarcoma oncogene homolog (sMaf) proteins to activate stress-dependent expression of a cluster of cytoprotective genes involved in the regulation of metabolism, detoxication, and redox balance via cis-acting antioxidant/electrophile response elements (AREs) (Figure 3) [58,59,60]. Rapamycin^1^ has been reported to attenuate the paraquat (PQ)-induced PF by promoting Nrf2 translocation to the nucleus and enhancing the expression of Nrf2 [61]. Vitamin U ameliorates lung toxicity by binding to the ETGE motif to promote Nrf2 dissociation from the Nrf2/Keap1 complex and translocation to the nucleus [62]. Notably, some small molecules can interfere with Keap1-mediated Nrf2 ubiquitination and degradation, causing the accumulation of Nrf2. For example, in the process of inhibiting PF, tanshinone IIA^2^ [63] and pterostilbene^3^ [64] regulate redox homeostasis by impairing the binding of Keap1 with Nrf2 and maintaining Nrf2 stability.

Currently, the downstream genes of Nrf2 can be divided into three categories according to their functions: cellular antioxidants, such as glutathione peroxidase (GPx), thioredoxin (Trx), thioredoxin reductase (TrxR), peroxiredoxin (Prx), and heme oxygenase-1 (HO−1); phase II detoxifying enzymes, including glutathione S-transferase (GST), NAD(P)H quinone oxidoreductase 1 (NQO1), superoxide dismutase (SOD), and CAT [65,66]; and transporters, such as multidrug resistance-associated protein (MRP) [67]. The above gene expression products can effectively regulate intracellular levels of reactive oxygen species (ROS), protect macromolecules from oxidative and xenobiotic damage, and reduce the toxicity of exogenous substances. Hence, the critical role of Nrf2 manifests in regulating oxidative stress and suppressing the inflammatory response. Researchers have therefore set their sights on Nrf2 and attempted to unravel its complex role in PF.

## 3. Nrf2 and Inflammation in Pulmonary Fibrosis

Endogenous and exogenous poisons, such as free radicals, air pollutants, and chemicals, attack the lungs, causing multiple inflammatory events due to their unique anatomical location. There is interplay between various cell types, including alveolar epithelial cells, fibroblasts, endothelial cells, and inflammatory cells (e.g., neutrophils, macrophages, lymphocytes, and eosinophils), whose injury recruits a fibrotic response, implicating that the occurrence and development of PF is a dynamic process [4]. Neutrophils and eosinophils are mainly involved in the acute phase of lung injury, whereas lymphocytes and macrophages govern chronic inflammation [68,69]. In the early stage of PF, many inflammatory cells infiltrate in the alveoli, releasing pro-inflammatory proteins and pro-inflammatory cytokines, such as tumor necrosis factor (TNF)-α, interleukin (IL)-1β, and IL-6, which are considered the primary cause of lung tissue scarring [70,71]. Etiologically, sustained inflammatory responses produce several significant ROS, resulting in direct or indirect injury to alveolar epithelial cells [72]. Damaged bronchial and alveolar epithelial cells and other resident cells further exacerbate various inflammatory mediators, proteases, and ROS release, which boost the recruitment of inflammatory cells and aggravate collagen accumulation in the lung tissue [73]. Alveolar epithelial cells are composed of alveolar type I (AT1), through which gas exchange takes place, and AT2 cells, which generate a large amount of surfactant. AT2 cells, which are considered as alveolar stem cells and can differentiate into AT1 cells [74], represent underlying initiating mechanisms responsible for PF in response to repetitive lung damage [75]. The functional impairment of AT2 cells and the development of a pro-fibrotic phenotype play critical roles in driving IPF. A recent study showed that loss of Cdc42 function in AT2 cells exposed to elevated mechanical tension promoted periphery-to-center progressive lung fibrosis [76], providing support for this hypothesis. EMT in alveolar epithelial cells, a key step in PF, will be discussed in detail later.

Indeed, innate immune cells, alveolar macrophages, and principally monocyte-derived alveolar macrophages play an important role in the formation of PF. When harmful agents attack the lungs, alveolar macrophages are the first line of defense to initiate inflammatory reactions and boost the infiltration of neutrophils [77,78]. Studies have shown that two distinct subsets of macrophages can be found in the lungs, tissue-resident alveolar macrophages and monocyte-derived alveolar macrophages, in a mouse model of bleomycin (BLM)-induced PF [79]. They also causally implicated that specific deletion of monocyte-derived alveolar macrophages reduced asbestos-induced fibrosis severity [80].

The transcription factor Nrf2 is known to attenuate inflammatory reactions [81,82]. Cho et al. reported that Nrf2^−/−^ mice were more vulnerable to BLM-induced inflammation and PF than wild-type mice and ascertained the protective effects of Nrf2 [83]. In mice lacking Nrf2, the resolution of lung injury and inflammation is compromised [84]. In irradiated Nrf2 null mice, AT2 cell loss and the corresponding development of PF were potentiated [85]. Nrf2 is believed to be required for alveolar macrophage-mediated apoptotic neutrophil clearance [86]. A single exposure of mouse lungs to zinc oxide nanoparticles increased the number of total cells, including macrophages, lymphocytes, neutrophils, and eosinophils, in bronchoalveolar lavage fluid (BALF) both in wild-type mice and Nrf2^−/−^ mice, but Nrf2^−/−^ mice expressed a greater increase [87]. In addition, when exposed to multiwalled carbon nanotubes, Nrf2 knockout (KO) mice displayed apparent pulmonary infiltration of granulocytes, macrophages, and B and T lymphocytes [88].

**Figure 4:**
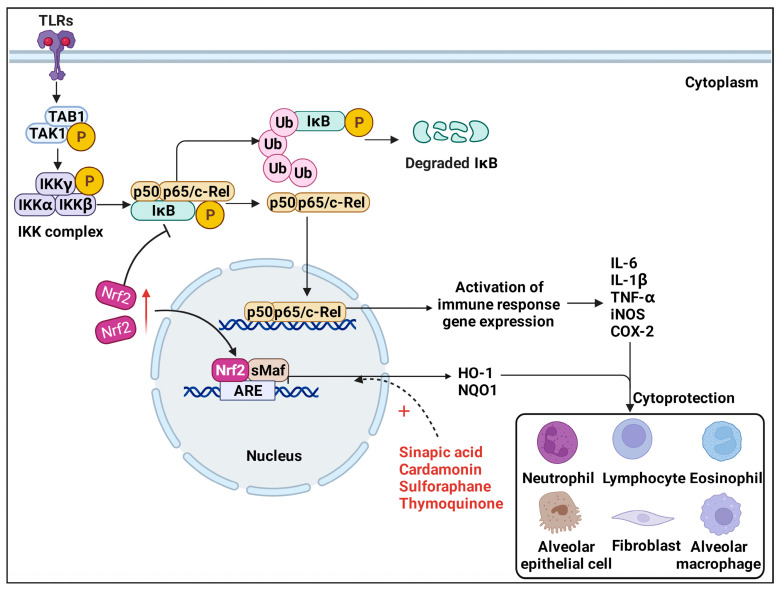
**Nrf2/HO−1 pathway and TLRs/NF-κB pathway.** In canonical TLRs/NF-κB signaling, TLRs activate TAK1-TAB1 kinase complex, resulting in IKK complex-meditated release of NF-κB dimers and phosphorylation of IκB. Phosphorylated IκB frees NF-κB dimers and permits NF-κB dimers translocated into the nucleus. However, activation of Nrf2 can inhibit IκB phosphorylation in the canonical NF-κB pathway, thereby reducing the nuclear accumulation of NF-κB dimers and inhibiting its downstream immune response genes expression, such as IL-6, TNF-α, IL-1β, iNOS, and COX-2. Moreover, elevated expression of HO−1 mediated by Nrf2 also demonstrated significant anti-inflammatory and inhibition of apoptosis effects in the progression of PF.

Several cellular and molecular mechanisms related to Nrf2 have been proven to be involved in the advancement and resolution of lung inflammation (Figure 4).

### 3.1. TLRs/NF-κB Pathway

Nuclear factor-κB (NF-κB) is a dimeric multifunctional nuclear transcription factor composed of p50 and p65 subunits, which are transferred from the cytoplasm to the nucleus when activated. Extensive biological activities, including the transcription of various cytokines (IL-6, TNF-α, and iNOS), adhesion molecules (COX-2), and chemokines, can be promoted by NF-κB, which is considered a prototypical pro-inflammatory signaling pathway [89]. It has been reported that Nrf2-Keap1 attenuates IκBα phosphorylation in the canonical NF-κB activation pathway, thereby reducing the nuclear accumulation of NF-κB [90]. During canonical NF-κB signaling, the release of canonical NF-κB dimers is controlled by the inhibitor of kappa B kinase (IKK) complex, which consists of IKK1 (IKK α), IKK2 (IKK β), and NEMO (IKK γ) [91]. Transcriptional activation of NF-κB occurs when the IKK complex is activated by transforming growth factor (TGF)-β1-activated kinase 1 and the TAK1-binding protein 1 (TAK1-TAB1) kinase complex, resulting in phosphorylated IkB and its later degradation [92,93]. Phosphorylation of IκBα frees NF-κB and permits NF-kB dimers to enter the nucleus [94]. Hence, Nrf2-mediated inhibition of IκBα phosphorylation and NF-κB translocation to the nucleus might be effective treatment options. Treatment with pirfenidone suppressed chronic intermittent hypoxia-augmented lung fibrosis in BLM-treated mice by upregulating Nrf2 and downregulating NF-κB [95]. Another study showed that, through inhibition of apoptosis and induction of Nrf2/HO−1-mediated antioxidant enzymes by means of suppressing NF-κB signaling, sinapic acid^4^ ameliorates BLM-induced lung fibrosis in rats [96].

### 3.2. Nrf2/HO−1 Pathway

Heme oxygenase-1 (HO−1) is the inducible, rate-limiting enzyme in the catabolism of heme, catalyzing the breakdown of heme into iron, biliverdin, and carbon monoxide [97]. HO−1 is one of the classic genes controlled by Nrf2 [98]. Elevated expression of HO−1 mediated by Nrf2 demonstrated significant anti-inflammatory and inhibition of apoptosis effects in the progression of PF. The latest research shows that cardamonin provokes the Nrf2/HO−1 axis in alveolar macrophages and exhibits anti-inflammatory and antioxidative effects on phorbol 12-myristate 13-acetate-induced pulmonary inflammation [71]. Thymoquinone^5^ targeting the Nrf2/HO−1 signaling pathway abrogates the inflammatory response in BLM-induced PF in rats [99]. Additionally, dihydroartemisinin^6^ regulated the oxidative stress process through the Nrf2/HO−1 signaling pathway in BLM-induced PF model [100]. Atractylenolide III^7^ attenuates BLM-induced experimental PF through the Nrf2/NQO1/HO−1 pathway [101]. The polysaccharide FMP-1 has been noted to attenuate cellular oxidative stress and protect alveolar epithelial cells through the PI3K/AKT/Nrf2/HO−1 pathway [102]. These observations indicate that Nrf2 is essential for the control of inflammation. Previously, it was generally believed that Nrf2 suppresses inflammation by controlling redox levels. More recent research has shown that Nrf2 also plays a part in controlling the expression of inflammatory cytokines. Nrf2 disturbs LPS-induced transcriptional upregulation of pro-inflammatory cytokines, such as IL-6 and IL-1β [103]. Nrf2 has also been reported to attenuate inflammation by suppressing Toll-like receptor (TLR)4 and Akt signaling [104].

## 4. Nrf2 and Oxidative Stress in Pulmonary Fibrosis

The triggering process of PF is multifactorial, and accumulating evidence indicates that oxidative stress is still a key player in the pathogenesis of PF [105,106]. Given the high level of oxygen to which the lungs are exposed, the lungs are more sensitive to oxidative stress than other tissues [107]. Oxidative stress stimulates an imbalance between oxidants/antioxidants, which significantly contributes to lung fibrosis [108]. Exogenous (air pollution, cigarette smoke, silica particles) and endogenous oxidants (mitochondrial ROS, hydrogen peroxide, superoxide anions, and NO) attack alveolar epithelial cells, pulmonary vascular endothelial cells and lung macrophages and induce the formation of ROS and reactive nitrogen species (RNS) [10,12,61,109]. ROS are important oxidative stress markers, and oxidative stress usually arises from the overproduction of ROS. ROS may damage cellular macromolecules, including DNA, lipids, and lesioned proteins, which perturb normal cell signaling pathways and cause irreversible dysfunction and apoptosis [110]. It is well known that silica can effectively increase the production of ROS in airway epithelial cells and lead to PF [111]. In recent years, a link between the inhalation of crystal-line silica and PF has been reported [112]. Tanshinone IIA has been reported attenuate silica-induced PF via activation of the Nrf2/Trx/TrxR axis and Nrf2-mediated inhibition of NOX4 expression, EMT, and TGF-β1/Smad signaling [113,114,115]. These findings indicate that Nrf2 plays an important role in protecting against silica-mediated oxidative stress and PF. Moreover, the inflammatory state is possibly elevated by oxidative stress via the activation of NF-kB, subsequently activating and recruiting immune cells (macrophages and T-cells) [116]. These inflammatory cells further irritate free radical production, including hydroxyl radicals and superoxide radicals [117], resulting in a decrease in classic antioxidant enzymes, including superoxide dismutase (SOD), catalase, and glutathione.

### 4.1. Nrf2 Downstream Antioxidant Products

Previous evidence has shown that the Keap1-Nrf2 pathway regulates ROS production through mitochondria and NADPH oxidase [118]. Nrf2 and its target genes (e.g., HO−1 and NQO1) can protect normal cells from oxidative stress and readily eliminate ROS. Melatonin has been reported to activate the Nrf2 signal transduction key antioxidant target genes HO−1 and NQO1 by increasing Sirtuin1 (SIRT1) expression and peroxisome proliferator-activated receptor coactivator-1α (Pgc-1α) deacetylation, defending against Cr(VI)-induced pulmonary injury [119]. Nrf2 regulates classical antioxidant enzymes, including SODs, catalase, GPx, and GSH reductase, directly inactivating ROS and preventing ROS-initiated reactions, while the phase 2 detoxifying enzymes GSTs and NQO1 play an indirect role by promoting the excretion of oxidative and active secondary metabolites and the biosynthesis/cycling of thiols [120]. For instance, Nrf2 directly enhanced the expression of the antioxidant proteins Trx and TrxR, promoted by sodium tanshinone IIA sulfonate (STS), and has been proven to attenuate silica-induced PF [113]. Rosavin^8^ activates the Nrf2 pathway to inhibit the occurrence of oxidative stress and enhance MDA, SOD, and GSH-Px expression [121]. The therapeutic roles of vitamin D_3_^9^ in the treatment of particle-associated PF also by activating Nrf2 signaling and promoting the expression of its downstream antioxidant products [122].

### 4.2. Nrf2/NOX4 Pathway

Mitochondria are suspected to be the main site of intracellular ROS generation, and NADPH oxidase (NOX), especially NADPH oxidase-4 (NOX4)-mediated superoxide production, is the main nonmitochondrial source of ROS accumulation [123,124,125]. In the NOX family (NOX1, NOX3, NOX4, NOX5, DUOX1, and DUOX2) [126], most NOX enzymes can catalyze the reduction of molecular oxygen to superoxide (O_2_^−^), but NOX4 catalyzes the reduction of molecular oxygen to H_2_O_2_ [127]. NOX4 also potentiates myofibroblast activation in response to TGF-β1, which seems to be a key factor in promoting fibrosis [128,129]. Therefore, therapeutic targeting of the Nrf2/NOX4 pathway to alleviate oxidative stress appears to be an effective option. Research has shown that polydatin protects against ROS-induced PF and reverses TGF-β1-induced pulmonary epithelial cell EMT in asthma by promoting Nrf2-mediated expression of HO−1 and NQO1 and inhibiting NOX1 and NOX4 expression [130]. S-Allylmercaptocysteine^10^ [131] and a gallic acid derivative^11^ [132] exhibited antioxidative capacity by influencing the TGF–β1/Smad and NOX4/Nrf2 pathways and increasing the expression of antioxidants, such as HO−1, GSH, and SOD. Ethyl acetate extract of salvia miltiorrhiza^12^ (EASM) [133] and tanshinone IIA [115] have been reported to upregulate Nrf2, and downregulating NOX4 positively alleviated oxidative stress in mice.

**Figure 5:**
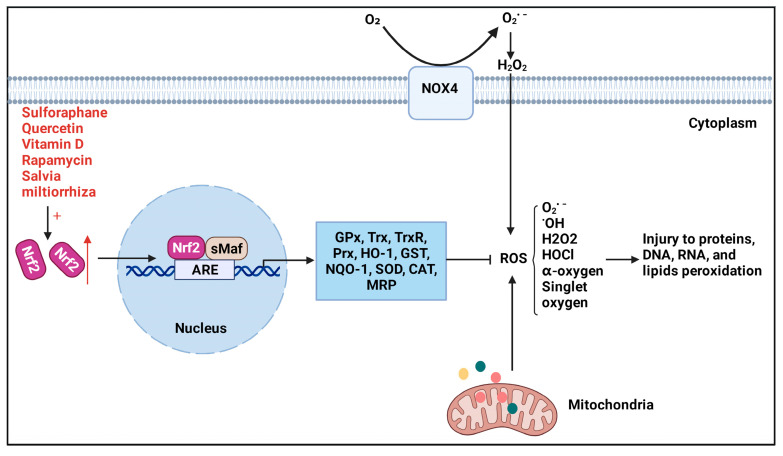
**Nrf2 downstream antioxidant products and Nrf2/NOX4 pathway.** Various Nrf2 activators can effectively promote Nrf2 entry into the nucleus and lead to increased expression of its downstream antioxidant products, such asHO−1and SOD, which can further eliminate potential damage to DNA, RNA, proteins, and lipid peroxidation caused by mitochondrial-derived and NOX4-derived ROS.

In addition, it has been reported that the intrinsic mechanism by which itaconate facilitates the transition of the macrophage phenotype from M1 to M2 presumably covers the activation of the Nrf2-Keap1 pathway and the deterrent of ROS [134]. In general, due to the central role Nrf2 plays in ROS detoxification (Figure 5), Nrf2 is an attractive therapeutic candidate for the pharmacological protection of PF.

## 5. Nrf2 and Fibroblasts in Pulmonary Fibrosis—TGF-β1/Smad Pathway

**Figure 6:**
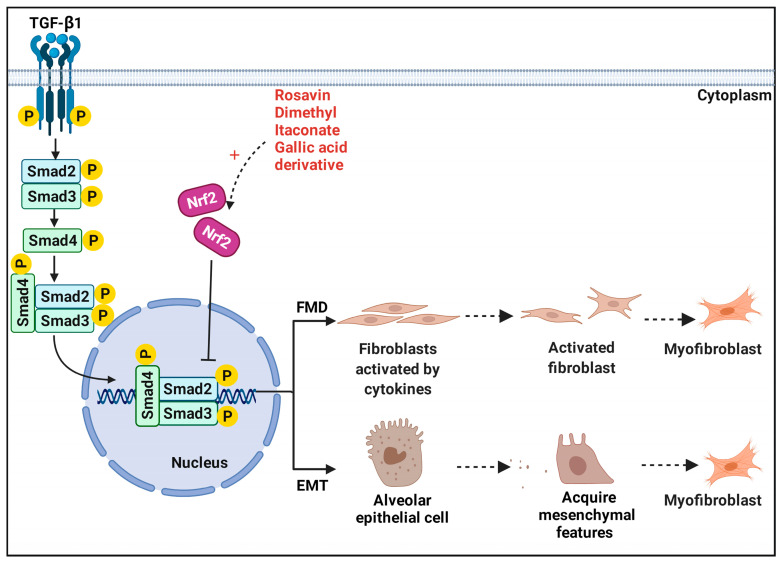
**Nrf2 and TGF-β1/Smad pathway.** Under pathological conditions, TGF-β1 can be produced by a wide variety of cell types, including alveolar macrophages, neutrophils, alveolar epithelial cells, endothelial cells, fibroblasts, and myofibroblasts. Abnormally activated TGF-β1/Smad pathway induced disturbances of the homeostatic microenvironment are critical to promote FMD and EMT processes. However, some compounds, such as rosavin and itaconate can specifically block these two processes by activating Nrf2 and provide protection against pulmonary fibrosis.

Aberrant activation and proliferation of myofibroblasts appear to be positively related to the pathogenesis of PF (Figure 6) [135,136], which can synthesize pro-fibrotic proteins including α-smooth muscle actin (α-SMA), collagen I and III, and fibronectin, finally resulting in excessive secretion and accumulation of ECM [137,138].

### 5.1. Fibroblast–Myofibroblast Differentiation

TGF-β1 has been identified as a major pro-fibrogenic cytokine in IPF patients and can be produced by a variety of cells, such as alveolar macrophages, alveolar epithelial cells, and myofibroblasts [139,140]. Recently, a growing number of studies have suggested that fibroblast–myofibroblast differentiation (FMD) is a critical cellular phenotype during the occurrence and deterioration of PF [141,142,143]. FMD can increase with an elevated level of ROS in fibroblasts under oxidative stress. Moreover, FMD is known as the primary source of myofibroblast accumulation [144,145]. Under pathological conditions, lung fibroblasts are irritated by TGF-β1 and stress, triggering differentiation into myofibroblasts and eventually leading to PF [146]. Consequently, FMD is an important therapeutic target for PF. Studies have shown that Nrf2 has a protective effect during PF and can inhibit the FMD process. Compared with Nrf2 knockdown, Nrf2 activation increased antioxidant capacity and myofibroblast dedifferentiation in IPF fibroblasts [147]. The activation of Nrf2 by dimethyl itaconate^13^ can protect against TGF-β1-induced FMD via the ROS/TXNIP signaling pathway and inhibit TXNIP-mediated FMD in PF [141]. Tanshinone IIA inhibited myofibroblast activation, rebalanced Nrf2-NOX4, and limited glutaminolysis in myofibroblast proliferation by activating the Nrf2/GSH signaling pathway [63].

### 5.2. Epithelial–Mesenchymal Transition

The antifibrotic function of Nrf2 is also embodied in the suppression of EMT and TGF-β1/Smad signaling [109,130,148]. EMT is considered to be a convertible process in the evolution of PF, during which epithelial cells gradually acquire mesenchymal features, such as the mesenchymal marker α-smooth muscle actin (α-SMA), and lose the epithelial adhesion protein E-cadherin (E-cad) [30,149]. The dysregulation between the alveolar epithelium and its associated mesenchyme lead to unchecked proliferation of extracellular matrix-producing cells [150]. It is widely recognized that EMT is another major source of myofibroblasts [151]. The TGF-β1/Smad signaling pathway promotes EMT and regulates the expression and irreversible deposition of ECM proteins, such as collagen I, fibronectin, and α-SMA [152,153]. Previous evidence has shown that Nrf2 alleviates PF by blocking EMT progression [154]. In addition, Nrf2 attenuated TGF-β1-induced EMT with downregulation of high-mobility group box 1 (HMGB1), a transcription factor-like protein and novel mediator of EMT [30]. Nrf2-mediated Tan IIA copes with silica-induced oxidative stress, EMT, and TGF-β1/Smad pathway inhibition [114]. Through the phosphoinositide 3-kinase (PI3K)/GSK-3β axis activating the Nrf2-dependent antioxidant pathway, melatonin attenuated LPS-induced EMT [155]. Similarly, activating the Nrf2-dependent antioxidant pathway successfully alleviated the evolution of EMT by regulating the aberrant expression of Numb, a phosphotyrosine-binding domain (PTB) protein, implicated in EMT [148].

## 6. Potential Therapies and Nrf2 Activators

In general, recurrent damage to susceptible individuals’ lungs will promote pro-oxidant, pro-inflammatory, and pro-fibrotic process microenvironments [156]; these regulatory processes do not operate independently but commonly interfere with each other. Oxidative stress and ROS production are related to the activation and production of many of inflammatory cytokines and pro-fibrotic growth factors [157], such as TGF-β1, in turn, promote ROS formation primarily by inducing the expression and activity of NOX4 in various cell types [158]. Evidence has clearly shown that oxidative stress and inflammation are the driving forces of myofibroblast activation [159]. Mechanistically, the Nrf2 signaling pathway plays a crucial role in these processes; thus, Nrf2-dependent antifibrosis therapies are vital for the treatment of PF. Three different mechanisms, as mentioned above, have been demonstrated to account for the activation of Nrf2: Keap1-dependent, Keap1-independent, and other regulators. Hence, targeting Nrf2 activators to find feasible future treatment options for PF patients has been a proven perspective in recent studies. Scientists have discovered many natural or synthetic Nrf2 agonists that may be effective in treating PF (Table 1).

Itaconate, an endogenous metabolite from the tricarboxylic acid cycle, was recently reported to have notable anti-inflammatory and immune-regulated effects [160]. It has been demonstrated that itaconate directly modifies 151, 257, 288, 273, and 297 on the protein Keap1 via alkylation of cysteine residues enabling Nrf2 to increase the expression of downstream antioxidant and anti-inflammatory genes [161]. Dimethyl itaconate (DMI), a cell-permeable itaconate derivative synthesized in vitro, was reported to protect against PF via activating Nrf2 and inhibiting thioredoxin-interacting protein (TXNIP) expression, thereby restraining TXNIP-mediated FMD [141]. Collectively, itaconate is a crucial anti-inflammatory metabolite that acts via Nrf2 to limit inflammation and control the severity of PF [161,162,163].

Rapamycin (sirolimus) is a potent inhibitor of the mammalian target of rapamycin (mTOR) which has been increasingly used to help organ transplant recipients prevent graft rejection [164]. The potential value of rapamycin in PQ-induced and TGF-α–induced PF has been proved [165,166]. Studies have shown that rapamycin protects against PQ-induced PF by activating the Nrf2 signaling pathway and inhibiting the EMT process [167]. A recent report also supports this notion by showing that rapamycin could inhibit PQ-induced oxidant stress and enhance the expression of Nrf2 [61]. However, the exact mechanism of rapamycin regulating the expression of Nrf2 needs further study.

Sulforaphane^14^ (SFN), a *Brassica oleracea* extract, is a well-studied potent activator of Nrf2 which has been previously reported to prevent BLM-induced PF in mice via Nrf2 activation [168]. The anti-fibrotic function of SFN was largely dependent on LOC344887, a long noncoding RNA, which presents a novel therapeutic axis for the prevention and intervention of PF [8]. Other natural products, such as Bletilla Striata^15^ [10], Sarcodon aspratus^16^ [169], Arenaria kansuensis^17^ [170], quercetin^18^ [171], chelerythrine^19^ [172], and bergenin^20^ [173] can all protect against PF through the Nrf2-dependent mechanism. Except for a single botanical drug, Chinese herbal formulas including Jinshui Huanxian formula^21^ (JHF) which is composed of 11 medicinal herbs also exhibit protective effects on PF [174].

In addition, dimethyl fumarate^22^ which has been approved for the first-line treatment of relapsing-remitting multiple sclerosis [175]; dihydroartemisinin which is traditionally used to treat malaria [100]; chloroquine^23^ which has been used for malaria treatment [176]; and proton pump inhibitors such as esomeprazole^24^ [156] have all been shown to inhibit the progression of PF by Nrf2 signaling. Thus, there is great potential for translating these drugs rapidly into clinical practice for treatment of PF.

In order to overcome the physiological barriers and prolong the treatment time of drug in the lungs, local drug administration, such as inhalation, can provide drugs directly to lung lesions and reduce the accumulation of drugs in other organs and improve therapeutic efficacy. However, inhaled particles can be swept out of the lung by cilia in the trachea and bronchi or phagocytosed by tissue-resident alveolar macrophages, which significantly affects their therapeutic effect. Recent evidence suggests that the application of nanotechnology is expected to solve these problems. Liu et al. designed a dimethyl fumarate-loaded ROS responsive liposome as an inhaled drug which presented enhanced antifibrotic effect, compared with direct dimethyl fumarate instillation [175]. This ROS-responsoftenive liposome is clinically promising as an ideal delivery system for inhaled drug delivery.

**Table 1:** Potential therapies and Nrf2 activators for the treatment of pulmonary fibrosis.

Compound	Model	Target	Function and Detection Index	Refs
Rapamycin^1^	PQ-treated male rats and LFs	Nrf2 activating	Suppressed PQ-induced oxidant stress, cell death and apoptosis, fibrosis-related factors, reversed PQ-induced FMD and PF induced by PQ.	[61]
Tanshinone IIA^2^	Silica-treated silicosis rat and NIH-3T3 cells	TGFβ1/Smad Nrf2/ NOX4 Nrf2/GSH	Reduced the levels of collagen deposition, TGF-β1, α-SMA, Col-I, Col-III, NOX4, ROS; increased the levels of Nrf2, HO−1, NQO1, Gclc, Gclm, and GSH; regulated myofibroblast activation, protected Nrf2 from protein ubiquitination, promoted Keap1 degradation.	[113,114,115]
Pterostilbene^3^	Lps-treated female BALB/C mice	Nrf2 activating	Decreased lung injury and fibrosis scores; reduced levels of Col-I, TGF-β1, HYP, IL-1β, IL-6, TNF-α; increased the levels of Nrf2, HO−1, NQO1, GSH, SOD.	[64]
Sinapic acid^4^	BLM-treated SD rats	Nrf2/HO−1 NF-κB	Increased the levels of Nrf2, inflammatory cell population, GPx, CAT, Bcl-2; reduced the levels of MDA, TNF-α, IL-1β, MPO, MMP-7, HYP, TGF-β1, NF-κB; restore the antioxidant system, inflammatory or fibrotic alterations.	[96]
Thymoquinone^5^	BLM-treated Wistar rats	Nrf2/HO−1	Decreased levels of HYP, LDH, total and differential leukocytes, MDA, TNF-α, IL-1β, MPO, MMP-7, caspase-3, Bax, NF-κB; upregulate Nrf2, HO−1, Bcl2; ameliorated severe hemorrhage, thickening of alveolar septa, emphysema, infiltration of leukocytes in walls alveoli and fibroplasia, inflammation, and PF.	[99]
Dihydroartemisinin^6^	BLM-treated SD rats and AECs	Nrf2/HO−1	Reduced the levels of α-SMA, MDA; increased the levels of E-cadherin, Nrf2, HO−1, SOD, and GSH; mitigated alveolitis severity, relieved fibrosis scores, inhibited the increase in the myofibroblasts–like processes of the AECs.	[100]
Atractylenolide III^7^	BLM-treated SD rats	Nrf2/NQO1/HO−1	Reduced the expression of Caspase-3 and Caspase-9, IL-6, iNOS, TNF-α, MDA, LDH; upregulated the levels of Nrf2, NQO1, HO−1, SOD, GSH, IL-10; improved lung function alleviated PF and oxidative stress.	[101]
Rosavin^8^	BLM-treated Kunming mice	Nrf2/NF-κB TGF-β1	Inhibited inflammatory cells, MDA, HYP, NF-κB-p65, α-SMA TGF-β1 levels; improved Nrf2, SOD, GSH-Px levels; ameliorated PF, alveolar inflammatory cell contents.	[121]
Vitamin D_3_^9^	Particles-treated Nrf2^+/+^ and Nrf2^−/−^ C57BL/6 mice, HFLIII cells	Nrf2 activating	Reduced the levels of α-SMA, FN, E-cadherin; increased the levels of N-cadherin, Nrf2, VDR; limited fibroblast cells’ migration, FDM, ECM.	[122]
S-Allylmercaptocysteine^10^	BLM-treated C57/BL6 mice	Nrf2/NOX4 TGF-β1/Smad	Increased antioxidants such as HO−1, GSH, and SOD; decreased HYP, SMA; ameliorated the pathological structure, and decrease inflammatory cell infiltration and pro-inflammatory cytokines in BALF.	[131]
Gallic acid derivative (GAD)^11^	BLM-treated C57/BL6 mice	Nrf2/NOX4 TGF-β1/Smad	Reduced the levels of α-SMA, HYP, collagen type I/III, IL-6, TGF-β1, NOX4; increase the levels of SOD and GSH; increased body weight, survival rate, and alleviated alveolar structure, alveolar inflammation, and the degree of PF.	[132]
Salvia miltiorrhiza^12^	BLM-treated C57/BL6 mice and NIH-3T3 cells	Nrf2/GSH Nrf2/Keap1 Nrf2/Nox4	Reduced the levels of TGF-β1, α-SMA, ECM, COL-1, NOX4, ROS, PKC-δ, Smad3; increase the levels of Nrf2, NQO1, HO−1; protected Nrf2 from protein ubiquitination, PF; regulated myofibroblasts activation, Increased the sensitivity of fibroblasts to the loss of GSH.	[133]
Dimethyl itaconate^13^	TGF-β1-induced FMD in vitro and BLM-treated mouse	Nrf2 activating	Nrf2 decreased TXNIP expression and alleviated FMD in PF; Nrf2 inhibited TGF-β1-induced FMD and the increase of ROS.	[141]
Sulforaphane^14^	BLM-treated C57/BL6 mice	Nrf2 activating	Reduced the levels of caspase-3, IL-1β, TNF-α, TGF-β, HYP, 3-NT, and 4-HNE; increased the levels of Nrf2, HO−1, NQO1, SOD1, and CAT; alleviated BLM-induced alveolar epithelial cell apoptosis, alveolitis, collagen accumulation, lung oxidative stress, and lung fibrosis.	[168]
Bletilla striata^15^	SiO_2_-treated C57BL/6 mice and A549 cells line	Nrf2/HO−1/γ-GCSc	Reduced the levels of MDA, ROS; increased the levels of γ-GCSc, Nrf2, SOD, HO−1; protective effect of lung injury, lung cell viability, apoptosis, and ROS accumulation.	[10]
Sarcodon aspratus^16^	BLM-treated Kunming mice and A549 cells	MAPK/Nrf2/HO−1 TGF-β1/MAPK TLR4/NF-κB	Reduced the levels of ROS, MDA, TNF-α, IL-1β, IL-6, CTGF, MMP-2, HYP, α-SMA, ECM, TLR4, MyD88, NF-κB-p65; increased the levels of GSH-Px, SOD, Nrf2, HO−1, CAT, Smad7; inhibited H2O2-induced cell apoptosis, oxidative stress, fibrosis, phosphorylation of JNK, ERK and P38, weight loss.	[169]
Arenaria kansuensis^17^	PQ-treated C57BL mice	TGF-β1/Smad NF-κB-p65 Nrf2/NOX4	Downregulated α-SMA, TGF-β1, TNF-α, IL-6, IL-1β1, HYP, ROS, collagen deposition, NOX4; upregulate Nrf2, SOD, and GSH; improved mice survival rate, body weight, lung pathological lesion, and the lung index.	[170]
Quercetin^18^	BLM-treated BEAS-2B cells	Nrf2 activating	Reduced the expression levels of ROS, TNF-α, and IL-8; increased Nrf2-ARE binding, HO−1, and γ-GCS; restored the disturbed redox balance and reduce inflammation.	[171]
Chelerythrine^19^	BLM-treated C57/BL6 mice	Nrf2/ARE	Reduced the expression levels of fibronectin, α-SMA, TGF-β, 4-HNE, and HYP; upregulated the levels of SOD, GSH, Nrf2, HO−1, and NQO1; alleviates collagen deposition, oxidative stress, and PF.	[172]
Bergenin^20^	BLM-treated C57/BL6 mice and NIH3T3 cells	p62/Nrf2	Decreased content of α-SMA, COL-1, HYP, ROS, MDA; increased the levels and activity of Nrf2, GSH, SOD, HO−1, NQO1; inhibited the TGF-β1 induced FDM, oxidative stress, and PF.	[173]
Jinshui Huanxian formula^21^	BLM-treated SD rats, MRC-5 cells and NIH-3T3 cells	Nrf2/NOX4 TGF-β1	Reduced the levels of TGF-β1, collagen deposition, HYP, α-SMA, COL-I, COL-III, MDA, MPO, NOX4, FN1; increased the levels of Nrf2, GSH, SOD, CAT, NQO1, HO−1; suppressed the increases of lung coefficient, TGF-β1-induced FDM, ROS production	[174]
Dimethyl fumarate^22^	BLM-treated C57/BL6 mice; RAW264.7 and NIH-3T3 cells coculture	Nrf2 activating	Attenuated macrophage activity and fibrosis in mice; promoted Nrf2 and HO−1 expression and suppress TGF-β and ROS production; reduced fibroblast-to-myofibroblast transition and collagen production by NIH-3T3 cells.	[175]
Chloroquine^23^	PQ-treated male C57BL/6 mice	Nrf2/NQO1/HO−1 TGF-β	Reduced the levels of TNF-α, IL-1β, IL-6, NO, iNOS, MDA, α-SMA, TGF-β; increased the levels of SOD, NQO1, Nrf2, HO−1; attenuated lung injury, oxidative stress, decreases protein, inflammatory cells.	[176]
Esomeprazole^24^	BLM- or TGF-β-treated PHLE cells and fibroblasts	MAPK/Nrf2/HO−1 DDAH/iNOS	Reduced the levels of DDAH, iNOS, IL-1β, IL-6, TNF-α, COL-I, COL-III, COL-V; increased the levels of HO−1, NQO1, Nrf2; downregulates pro-inflammatory and profibrotic molecules, collagen expression; activates MAPK via phosphorylation.	[156]

The superscript number in the upper right corner of the compounds is numbered according to the order in which the compounds first appeared in the main text.

## 7. Conclusions and Perspectives

PF is an intractable disease that has long plagued humans. Research continues to face challenges, as the exact mechanistic aspects of PF are not well understood. The pathogenesis and molecular mechanisms associated with PF include pathological inflammation, imbalanced oxidative stress responses, and abnormal activation of myofibroblasts. The key pathological processes include FMD, EMT, and ECM deposition. Alveolar epithelial cells, fibroblasts, endothelial cells, neutrophils, macrophages, lymphocytes, and eosinophils are involved in this process. Given the complex biological pathogenesis of PF, which is regulated by multiple signaling pathways and cytokines, targeting a single mechanism to address unmet clinical needs in PF seems unlikely to reverse the disease. The transcription factor Nrf2 coordinates the expression of more than 500 cytoprotective and metabolic genes in response to various stresses to restore cellular homeostasis [25]. Alterations in Nrf2 regulatory genes play fundamental roles in the pathogenesis of PF, and the signaling pathways include TLRs/NF-κB, MAPK/Nrf2/HO−1/NQO1, p62/Nrf2, Nrf2/NOX4, Nrf2/GSH, and TGF-β1/Smad. Due to this wide range of functions, it may be effective to consider Nrf2 activators in combination with currently available treatment options in the clinic. These natural products come from a wide range of sources and act on multiple pathways to exert their pathological effects, which are appropriate for the multisystem, multitarget pathogenesis of PF [177]. Moreover, most Nrf2 activators are natural products with extraordinary therapeutic effects and can be easily applied to the daily diet, reducing the high physical and psychological burden on patients and enhancing quality of life.

Recently, considering the challenges associated with conventional oral and intravenous routes of drug administration, local delivery of drugs via nanoparticle carriers to the lungs is an emerging area of interest. Nanotechnology-based inhalation drug delivery methods possess numerous advantages including (1) uniform distribution of the inhaled drug among the alveoli, (2) better solubilization of the drug, (3) reduced drug accumulation in other organs, (4) long-term drug release, (5) lesser side effects, and (6) improved drug internalization to the lung cells [178,179,180]. In addition, some natural products, such as bergenin [173] and tanshinone IIA [63] whose anti-fibrotic effects have been reported by using human lung fibroblasts (HFL-1) cells and human fetal lung fibroblast (MRC-5) cells, respectively. These natural products may have significant potential for clinical translation, but the preclinical studies or tests are needed.

PF is known to result in irreversible loss of lung function, and future research should focus on prevention rather than cure. Continued investigations of Nrf2-mediated cellular defense mechanisms and preventive effects may provide insights to cure PF.
